# Adeno-Associated Virus (AAV) Capsid Stability and Liposome Remodeling During Endo/Lysosomal pH Trafficking

**DOI:** 10.3390/v12060668

**Published:** 2020-06-20

**Authors:** Bridget Lins-Austin, Saajan Patel, Mario Mietzsch, Dewey Brooke, Antonette Bennett, Balasubramanian Venkatakrishnan, Kim Van Vliet, Adam N. Smith, Joanna R. Long, Robert McKenna, Mark Potter, Barry Byrne, Sanford L. Boye, Brian Bothner, Regine Heilbronn, Mavis Agbandje-McKenna

**Affiliations:** 1 Department of Biochemistry and Molecular Biology, Center for Structural Biology, McKnight Brain Institute, College of Medicine, University of Florida, Gainesville, FL 32610, USA; bklins5@gmail.com (B.L.-A.); saajanspatel@ufl.edu (S.P.); mario.mietzsch@ufl.edu (M.M.); dendena@ufl.edu (A.B.); bambaiwallah@gmail.com (B.V.); kimvanvliet2@gmail.com (K.V.V.); jrlong@mbi.ufl.edu (J.R.L.); rmckenna@ufl.edu (R.M.); 2 Department of Chemistry and Biochemistry, Montana State University, Bozeman, MT 59717, USA; dbrooke@uab.edu (D.B.); bbothner@montana.edu (B.B.); 3 Department of Chemistry, College of Liberal Arts & Sciences, University of Florida, Gainesville, FL 32611, USA; adam.n.smith.487@gmail.com; 4 Powell Gene Therapy Center Vector Core, University of Florida, Gainesville, FL 32610, USA; mpotter@peds.ufl.edu (M.P.); bbyrne@ufl.edu (B.B.); sboye@ufl.edu (S.L.B.); 5 Department of Ophthalmology, University of Florida, Gainesville, FL 32610, USA; 6 Institute of Virology, Charité Medical School, 12203 Berlin, Germany; regine.heilbronn@charite.de

**Keywords:** AAV, trafficking, capsid, liposomes, PLA_2_, thermostability, infectivity

## Abstract

Adeno-associated viruses (AAVs) are small, non-pathogenic ssDNA viruses being used as therapeutic gene delivery vectors for the treatment of a variety of monogenic diseases. An obstacle to successful gene delivery is inefficient capsid trafficking through the endo/lysosomal pathway. This study aimed to characterize the AAV capsid stability and dynamics associated with this process for a select number of AAV serotypes, AAV1, AAV2, AAV5, and AAV8, at pHs representative of the early and late endosome, and the lysosome (6.0, 5.5, and 4.0, respectively). All AAV serotypes displayed thermal melt temperatures that varied with pH. The stability of AAV1, AAV2, and AAV8 increased in response to acidic conditions and then decreased at pH 4.0. In contrast, AAV5 demonstrated a consistent decrease in thermostability in response to acidification. Negative-stain EM visualization of liposomes in the presence of capsids at pH 5.5 or when heat shocked showed induced remodeling consistent with the externalization of the PLA_2_ domain of VP1u. These observations provide clues to the AAV capsid dynamics that facilitate successful infection. Finally, transduction assays revealed a pH and temperature dependence with low acidity and temperatures > 4 °C as detrimental factors.

## 1. Introduction

Many viruses, enveloped and non-enveloped, DNA and RNA packaging, require acidification during trafficking for successful infection [1]. In general, an acidic pH has been associated with two major mechanisms utilized by these viruses for their entry into the cytosol and/or uncoating at the appropriate location. Firstly, a low pH can directly induce capsid modifications to facilitate the escape from the endosome/lysosome [1]. Secondly, the acidic endosomal environment can trigger pH-dependent proteases that act on the capsid to facilitate the release of the encapsulated genome [2,3]. The Adeno-associated viruses (AAVs) are pH-dependent, non-enveloped viruses belonging to the *Dependoparvovirus* genus of the *Parvoviridae* family [4]. For these, as well as other parvoviruses, it is known that an acidic environment is essential for externalization of the unique N-terminal region of the minor capsid viral protein 1 (VP1), VP1u. This contains a phospholipase A_2_ (PLA_2_) domain, which enables escape from the endo/lysosomal pathway en route to the nucleus for genome uncoating and replication [5]. It has also been observed that amino side chain rearrangements and DNA compaction occur in AAVs in response to low pH [6]. However, how these pH-induced changes trigger or relate to VP1u externalization or uncoating are unknown.

Amino acid substitutions of residues within the active site of the PLA_2_ domain sequesters the AAVs and other parvoviruses within endosomes and lysosomes [7,8]. The PLA_2_s resemble secretory PLA_2_s in their requirement of calcium for activity, which is consistent with their function in the high-calcium environment of the endosomes [8]. Phospholipase A_2_ enzymes catalyze the hydrolysis of lipids into fatty acids and lysophospholipids. Unlike phospholipids, which have a cylindrical-like shape, lysophospholipids have a cone shape that induces strain on the lipid bilayer [9]. In an effort to alleviate this strain and prevent rupture of the bilayer, the membrane undergoes positive curvature [9]. This is recognizable as the blebs that invaginate into the endosome. In the case of parvoviruses, continued PLA_2_ activity induces enough strain in the endosomal membrane to form transient, limited pores, which enables viruses to escape to the cytoplasm [10,11]. However, in contrast to other pore-forming viruses, extensive endosomal lysis has not been reported for the parvoviruses [10,11].

The PLA_2_ of AAV2 is reported to be most active at physiological pH 7, with reduced activity in acidic or basic conditions [12,13]. However, the decrease in PLA_2_ activity at acidic conditions appears counterintuitive to its reported role in the AAV lifecycle: the PLA_2_ function of the VP1u is necessary for virus escape from the acidic endosome/lysosome compartments. Interestingly, different trafficking pathways have been reported for different AAV serotypes [14]. It is thus possible that variations in the AAV trafficking route can help to explain differences in serotype transduction efficiency. For example, AAV8 shows the enhanced transduction of liver cells over AAV2 [15] and a key variation in their life cycle is that AAV2 reportedly escapes from the late endosome (~pH 5.5) while AAV8 escapes from the early endosome (~pH 6), where the PLA_2_ would be more active, and does not encounter the late endosome [16,17]. It has also been reported that AAV2 capsids that travel from the early endosome to the recycling endosome (~pH 6.5) are more infectious than capsids that travel from the early endosome to the late endosome [18]. Understanding the physical properties of the AAVs under the conditions encountered in the early and late endosomes as well as lysosomes, and how these affect the externalization of the VP1u domain and its lipid interactions, could thus provide clues to differences in AAV transduction efficiency.

In this study, the pH-dependent thermostability and VP1u externalization of four selected AAV serotypes, AAV1, AAV2, AAV5, and AAV8, were characterized, in addition to visualization of their interaction with liposomes. Furthermore, the pH and temperature dependence of infection was analyzed. These serotypes were chosen because they span three different phylogenetic clades and one clonal isolate, comprise two different viral species (*dependoparvovirus A and dependoparvovirus B*), and have different transduction efficiencies in a wide array of tissues: AAV1 is efficient in skeletal muscle, AAV2 has broad tropism, AAV5 targets the lungs, and AAV8 is efficient in the liver [19,20]. AAV1, AAV2, and AAV8 were shown to be most stable at pH 5.5 while AAV5 was most stable at pH 7.5. The temperature at which VP1u is externalized differed between the viruses, consistent with differences in capsid stability. The transduction efficiency of these viruses followed the same trend as the serotype stability at the pHs tested and was temperature-dependent, with room temperature (RT) and 37 °C, along with extreme acidity (pH 2.5), being the most detrimental. Finally, the physical interaction of the four AAV capsids with liposomes was observed following treatment at pH 5.5 or heat-shock at temperatures that externalized VP1u. While all four viruses extensively remodeled lipid membranes at pH 5.5, the heat-shock temperature required to attain membrane remodeling differed and followed the capsid stability trend. Taken together, this study’s observations show that the biophysical and dynamic properties of the AAVs differ, despite the conservation of structural properties and function, such as the PLA_2_ activity, and should be taken into consideration in comparative studies of their infection pathway and transduction efficiency.

## 2. Materials and Methods

### 2.1. Cell Culture

*Sf*9 cells and cell lines expressing Rep and Cap for rAAV production were cultivated in suspension culture under constant agitation with serum-free Sf-900 medium supplemented with 1× antibiotic-antimycotic (Gibco, Gaithersburg, MD, USA) at 27 °C. HEK 293 cells were cultivated as adherent monolayers at 37 °C and 5% CO_2_ in DMEM supplemented with 1× antibiotic-antimycotic and 10% (*v*/*v*) fetal bovine serum (Gibco).

### 2.2. Cloning

The AAV2 VP1 and VP2 start codons of pFBDVPm11 [21] were mutated to GCG codons by site-directed mutagenesis (Agilent, Santa Clara, CA, USA) to generate pFB-AAV2-VP3. This plasmid was used to generate a recombinant baculovirus utilizing the Bac-to-Bac system according to the manufacturer’s manual (Invitrogen, Carlsbad, CA, USA). AAV2 VP1u DNA was amplified by PCR using pIM45 [22] as the template and the VP1u Forward Primer–5′-ATATGCGATCGCCATGGCTGCCGATGGTTATCTTC-3′ and VP1u Reverse Primer–5′-GATTGTTTAAACCTTAACAGGTTCCTCAACCAGGC-3′. Phusion high-fidelity DNA polymerase (NEB, Ipswich, MA, USA) was used per manufacturer’s protocol. The PCR product was cloned into the plasmid pFN22K (HaloTag7) CMVd1 Flexi Vector using *Nco*I and *Pme*I restriction sites and T4 DNA ligase (Promega, Madison, WI, USA).

### 2.3. Production and Purification of AAV2 VP1u Polypeptide

The resulting plasmid (pVH4) from the AAV2 VP1u cloning was transformed into Mach1 T1R competent cells per manufacturers protocol (Invitrogen). The DNA sequence of this plasmid was confirmed by Sanger sequencing. For protein expression, pVH4 was transformed into BL21-AI competent cells per manufacturer’s protocol (Invitrogen). Transformants were grown overnight at 37 °C in LB media supplemented with 25 µg/mL kanamycin. The starter culture was diluted 1:100 in fresh LB medium with 25 µg/mL kanamycin until mid-log phase (OD_600_~0.4), protein expression was then induced by addition of l-arabinose to a final concentration of 0.2%. After incubation for 4 h, the cells were collected by centrifugation at 4000× *g* for 20 min at 4 °C. Cells were resuspended in purification buffer (50 mM HEPES (pH 7.5), 150 mM NaCl, 0.005% IGEPAL CA-630), treated with lysozyme (0.2 mg/mL) and 0.5 mg/mL DNase I on ice for 10 min, followed by three freeze–thaw cycles in a dry ice/ethanol bath and in an ice/water bath. Cell lysates were centrifuged at 10,000× *g* for 30 min at 4 °C. The supernatant was collected and purified using HaloLink Chromatography Resin (Promega). HaloLink Resin was equilibrated with a purification buffer and then added to the supernatant and incubated under rotation for 1 h at RT. The tube was then centrifuged at 1000× *g* for 5 min at 4 °C. The supernatant was discarded and the resin washed three times in purification buffer and centrifuged at 1000× *g* for 5 min at 4 °C. To elute the bound protein from the resin, it was cleaved using ProTEV Plus protease. The His-tagged protease was removed on Ni-NTA agarose resin columns (Qiagen). The flow-through contained pure AAV2 VP1u. The amino acid sequence of AAV2 VP1u was confirmed by LC-MS/MS. Phospholipase activity of AAV2 VP1u was confirmed using the sPLA_2_ assay kit (Cayman Chemical, Hilden, Germany). The AAV2 VP1u protein was dialyzed into a citrate-phosphate buffer pH 7.5, 150 mM NaCl for cellular assays.

### 2.4. Production of AAV VLPs

Recombinant baculoviruses were used to generate AAV virus-like particles (VLPs) in *Sf*9 insect cells, as previously described [23,24,25,26]. Briefly, *Sf*9 cells were infected with a P3 baculovirus stock containing the desired AAV capsid gene [26] at a multiplicity of infection (MOI) of five plaque-forming units. Cells were harvested 72 h post infection and pelleted by centrifugation in a JA-10 rotor at 3000 rpm for 15 m at 4 °C. The supernatants were removed, and pellets were resuspended in lysis buffer (50 mM Tris-HCl pH 8.0, 100 mM NaCl, 2 mM MgCl_2_, 0.2% Triton X-100) and stored at −20 °C until virus purification. The supernatants from the cell pellets were subjected to PEG precipitation to collect virus capsids secreted into the media during infection. A total of 10% (*w*/*v*) PEG 8000 was added to the supernatants followed by stirring overnight at 4 °C. The precipitate was collected by centrifugation in a JA-10 rotor at 10,000 rpm for 1.5 h at 4 °C. The PEG pellets were resuspended in a lysis buffer and stored at −20 °C until purification was conducted.

The AAV1, AAV2, AAV5, and AAV8 VLPs were extracted from *Sf*9 cell pellets by three rounds of rapid freeze–thaw cycles. Benzonase (Millipore, Burlington, MA, USA) was added (25 U/mL) after the last thaw step and the lysates were incubated at 37 °C for 30 min to degrade cellular DNA. The lysates were clarified by centrifugation in a JA-20 rotor (Beckman Coulter, Brea, CA, USA) at 10,000 rpm for 15 min at 4 °C.

### 2.5. Purification of AAV VLPs

The clarified lysates were loaded onto a 20% (*w*/*v*) sucrose cushion to remove cellular contaminates and pellet the VLPs. The cell lysate was pipetted into a 26.3 mL centrifuge tube (Beckman Coulter). A glass Pasteur pipette was used to gently layer 5 mL of 20% sucrose (*w*/*v* in TNET buffer, 50 mM Tris-HCl pH 8.0, 100 mM NaCl, 1 mM EDTA, 0.2% Triton X-100) underneath the lysate. The virus was pelleted through the cushion by ultracentrifugation in a Type 70 Ti rotor (Beckman Coulter) at 45,000 rpm for 3 h at 4 °C. The supernatant was removed and the pellet resuspended in 1 mL TNMT buffer (25 mM Tris-HCl pH 8.0, 100 mM NaCl, 2 mM MgCl_2_, 0.2% Triton X-100).

The resuspended AAV1, AAV5, and AAV8 VLPs samples were further purified by a sucrose step-gradient ultracentrifugation (5, 10, 15, 20, 25, 30, 35, and 40% (*w*/*v*) in TNMT buffer). The gradient was formed by layering 1.35 mL of each percentage in an ultra-clear ultracentrifuge tube (Beckman Coulter) with a glass Pasteur pipette. The resuspended virus pellet was loaded on top of each gradient and the tubes were centrifuged in an SW41 Ti rotor (Beckman Coulter) at 35,000 rpm for 3 h at 4 °C. The 20–25% sucrose fraction (which showed a blue band under white light) was extracted with a syringe and dialyzed into 20 mM Tris-HCl pH 8.5, 250 mM NaCl to remove the sucrose.

In contrast to the AAV1, AAV5 and AAV8 VLPs, the AAV2 VLPs were purified by a discontinuous iodixanol step-gradient (15–60%) centrifugation [27]. Solutions of 15, 25, 40, and 60% iodixanol were prepared using Optiprep^TM^ medium (Sigma-Aldrich, St. Louis, MO, USA). The 15% solution additionally contained 1 M NaCl, while the 25% and 60% solutions included phenol red to provide contrast. Gradients were made in 32.4 mL OptiSeal^TM^ tubes (Beckman Coulter), first by layering 7 mL of the 15% solution, then 5 mL of the 25%, followed by the 4 mL of the 40%, and lastly the 3 mL of the 60% density medium underneath, using a glass Pasteur pipette. The clarified cell lysate was loaded on top. The iodixanol gradients were centrifuged in a Type 70 Ti rotor (Beckman Coulter) at 69,000 rpm for 1 h at 4 °C. Tubes were punctured and fractionated. Sodium dodecyl sulfate-polyacrylamide gel electrophoresis (SDS-PAGE) was used to verify the presence of VPs in the fractions.

The 25/40% and 40% iodixanol fractions were further purified by a 5 mL prepacked HiTrap Q HP sepharose anion exchange chromatography column (GE Healthcare, Chicago, IL, USA). Prior to purification, the column was washed in an AKTA FPLC system (GE Healthcare) with 25 mL of Buffer A (20 mM Tris-HCl, 15 mM NaCl pH 8.5), followed by 25 mL of Buffer B (20 mM Tris-HCl, 500 mM NaCl pH 8.5). Afterwards, the column was equilibrated with 50 mL of Buffer A. Fractions from the iodixanol gradient were diluted 1:10 (25% fraction) or 1:1 (25/40 and 40% fractions) in Buffer A. Samples were loaded onto the column using a peristaltic pump at a flow rate of 1 mL/min. The column was then loaded onto the FPLC system and washed with 50 mL Buffer A. The virus was eluted off the column in 1 mL fractions by linearly increasing the concentration of Buffer B. At ~250 mM NaCl, the virus was eluted.

### 2.6. Production and Purification of rAAV-GFP

Recombinant AAV (rAAV) vectors packaging green fluorescent protein (GFP) or luciferase were produced using two different methods, stable *Sf*9 cell lines and triple-transfection in HEK 293 cells, respectively. For the production of rAAVs using the OneBac system [28] logarithmically growing *Sf*9 suspension cell lines for the expression of AAV1, AAV2, AAV5, and AAV8 were infected with the recombinant baculovirus Bac-rAAV-GFP (pTR-Bac-UF26) at a MOI of 5, and harvested after 72 h as described above.

For rAAV production in HEK 293 cells, cells were seeded on 15 cm cell culture dishes. At a confluency of ~75%, the old growth medium was replaced with 15 mL of fresh Dulbecco’s Modified Eagle Medium (DMEM). Afterwards, the plasmids pTR-UF3-Luciferase, pHelper, and pXR1 (for rAAV1), pXR2 (for rAAV2), pXR5 (for AAV5), or pXR8 (for AAV8) were mixed at an equimolar ratio (total: 40 µg/15 cm plate). The DNA mixture was diluted in OptiMEM (Gibco) to a volume of 1 mL per 15 cm plate. After adding 125 µL of polyethyleneimine (1 mg/mL), the solution was mixed, incubated at RT for 15 min and added dropwise to the HEK 293 cells. The following day, 5 mL of fresh DMEM was added to the plate. The cells were harvested after 72 h as described above.

Recombinant AAV vectors produced by either production system were purified by AVB affinity ligand columns (Thermo Fisher Scientific, Waltham, MA, USA). Prior to purification, the cleared freeze–thaw supernatants were diluted 1:1 in 1× TD buffer (1xPBS with 1 mM MgCl_2_ and 2.5 mM KCl). After equilibrating the chromatography column with ten column volumes of 1× TD buffer, the lysates were loaded onto the column at a flow rate of 0.5 mL/min. Washing of the column was performed with 20 mL of 1× TD buffer. The AAVs were eluted with 0.1 M sodium acetate, 0.75 M NaCl pH 2.5 and neutralized immediately with 1/10 volume of 1 M Tris-HCl pH 10.

### 2.7. Confirmation of purity of VLPs and rAAVs

All the AAV VLP and rAAV samples were accessed for purity and integrity using SDS-PAGE and negative stain electron microscopy (EM), respectively. Briefly, for SDS-PAGE analysis, a 10 μL sample was loaded onto SDS polyacrylamide gels. After running, the gel was stained with GelCode Blue (Thermo Fisher Scientific) to check for the presence of VP1 (87 kDa), VP2 (72 kDa), and VP3 (62 kDa). For negative stain EM, carbon-coated copper 400 mesh grids (Ted Pella, Redding, CA, USA) were glow discharged for 1 min using a PELCO easiGlow^TM^ glow discharge cleaning system prior to use. Five microliters of sample was pipetted onto the grids and allowed to adhere for 2 min. Grids were washed in three 15 μL droplets of diH_2_O for 5 s each, and excess diH_2_O was removed using filter paper. Grids were stained in two 15 μL droplets of 2% uranyl acetate for 10 s and excess stain was removed by blotting with filter paper. Grids were examined using an FEI Tecnai G2 Spirit transmission electron microscope operating at 120 kV accelerating voltage, and images were collected using a Gatan UltraScan 1000XP camera.

### 2.8. rAAV pH and Heat Screen

rAAV1-GFP, rAAV5-GFP, and rAAV8-GFP or AAV2-VLP samples were diluted to 2 × 10^11^ gp/mL or 20 ng/mL, respectively, in citrate-phosphate with 150 mM NaCl at pH 7.4, 6.0, 5.5, or 4.0. The samples were heated for 5 min and cooled to 4 °C. After cooling, the samples were adhered to EM grids and stained as described above.

### 2.9. Native Dot Immunoblot

rAAV-GFP samples were diluted to 2 × 10^10^ vector genome containing particles/mL (~2 ng/μL) in citrate-phosphate with 150 mM NaCl at pH 7.4, 6.0, 5.5, or 4.0. Samples were then heated in a Bio-Rad C1000 thermo cycler for 5 min and cooled to 4 °C. After cooling, 10 μL of the heated samples were loaded onto a nitrocellulose membrane by vacuum suction using a Minifold Dot-Blot apparatus (GE Healthcare). Membranes were blocked in 5% milk/Tween-PBS for 1 h at RT or overnight at 4 °C. Membranes were probed for intact capsids using ADK1a (AAV1) [29], A20 (AAV2) (ARP, Waltham, MA, USA) [30]), ADK5a (AAV5) [29], and ADK8 (AAV8) [31]; denatured capsids using B1 [30]; and the presence of VP1u using A1 (ARP) [30] in 1% milk/Tween-PBS for 1 h at RT. Unbound antibodies were removed by three washes in Tween-PBS (5 min each), after which the membrane was incubated with a secondary horseradish peroxidase (HRP)-linked anti-mouse IgG antibody (GE Healthcare). After 1 h incubation, the membrane was washed three times in Tween-PBS to remove unbound antibodies (5 min each). The membrane was incubated with Immobilon chemiluminescent substrate (Millipore) and imaged to detect the presence of intact capsids, denatured capsids, or VP1u.

### 2.10. Determination of the Thermal Stability of AAV Capsids

Differential Scanning Fluorimetry was used to determine the thermal stability of AAV1, AAV2, AAV5, and AAV8 VLPs. This method monitors the binding of the SYPRO orange dye to hydrophobic regions of proteins that are exposed during unfolding. For the comparative analysis of VLPs at different pH, 2.5 µL of each AAV serotype at 1 mg/mL were added to 20 µL Citrate Phosphate Buffer [CiPO_4_ (0.2 M Na_2_HPO_4_, and 0.1 M Citric acid pH 7.4, pH 6.0, pH 5.5, and pH 4.0)] and incubated at 4 °C for 30 min. In addition, 2.5 µL of 1%-SYPRO-Orange dye (Invitrogen) was added to each mixture to make a total reaction volume of 25 µL. The assays were run in a Bio-Rad CFX Thermocycler instrument with temperature ranging from 30 to 99 °C, and ramping at 0.5 °C per step using FAM (485 nm) for excitation and ROX (625 nm) for emission. The rate of change of fluorescence with temperature was recorded, and the thermal profile is output as –dRFU/dT versus temperature. The thermal profile is then inverted by multiplying with -1 and normalized by dividing the raw values of the profile by the peak dRFU/dT value for evaluation. The peak value recorded on the thermogram is the T_m_. A negative control of 22.5 µL of each buffer and 2.5 µL of SYPRO-Orange was included for each run. All experiments were performed in triplicate.

### 2.11. Production of Liposomes

Powdered 1-hexadecanoyl-2-(9Z-octadecenoyl)-sn-glycero-3-phospho-choline (POPC) lipids (Avanti Polar Lipids, Alabaster, AL, USA) were resuspended in 50 mM HEPES pH 7.5, 100 mM NaCl, 1 mM CaCl_2_ to a concentration of 1.3 mM. Resuspended POPC was extruded through an Avanti Mini-Extruder to produce unilamellar liposomes. Briefly, 1 mL of resuspended lipids were solubilized by 15 cycles of rapid freeze–thaws in liquid-nitrogen and a 45 °C water bath. Solubilized lipids were then extruded through a 0.1 μm polycarbonate membrane (Avanti Polar Lipids) 29 times using the Avanti Mini-Extruded apparatus. Extruded liposomes were stored at 4 °C for up to one week.

### 2.12. Analysis of AAV–Liposome Interactions

The rAAV1-GFP, rAAV5-GFP, and rAAV8-GFP or AAV2-VLP samples were dialyzed into 50 mM HEPES, 100 mM NaCl, 1 mM CaCl_2_ at pH 7.4. Experiments were carried out in a total reaction volume of 10 μL and a final virus concentration of 2 × 10^11^ gp/mL. Heating experiments were performed as described above, except that the 1 μL of virus was diluted in an 8 μL HEPES buffer for heating and cooling, followed by incubation with 1 μL of POPC for 5 min at RT. For the pH 5.5 assay, 1 μL virus was diluted in 0.8 μL 100 mM citric acid and a 7.2 μL HEPES buffer to achieve a pH of 5.5 and incubated with 1 μL POPC at RT for 5 min.

Negative control assays of liposome modification included the use of untreated virus with POPC, AAV2 VLPs comprised of VP3 only with POPC, POPC alone, and pH-treated POPC. Positive controls to assess lipid modification included the use of bee venom PLA_2_ (bvPLA_2_) with POPC, AAV2 VP1u alone with POPC, and heated AAV2 VP1u with POPC. All negative-stain EM grids were prepared and viewed as described above. Experiments were performed in triplicate.

### 2.13. Analysis of AAV Transduction Efficiency after Incubation at Different pHs and Temperatures

Purified and highly concentrated rAAV vectors (titer range: 5–6 × 10^13^ gp/mL) packaging the luciferase gene (rAAV1-Luc, rAAV2-Luc, rAAV5-Luc, and rAAV8-Luc) were incubated in citrate-phosphate with 150 mM NaCl at pH 7.4, 6.0, 5.5, 4.0 or 2.5 for 24 h at −80 °C, 4 °C, RT, and 37 °C. Prior to infection, 10 µL of the incubated viruses were diluted in 300 µL of DMEM and then added to the HEK 293 cells on a 24-well plate. The total amount of rAAV vectors used correspond to a MOI of 100K for AAV1, AAV5, and AAV8 or a MOI of 10K for AAV2. The infected cells were incubated for 48 h and the transduction efficiency of the individual vector samples was determined by the Luciferase Assay System (Promega) according to the manufacturer’s instructions. Experiments were performed in triplicate.

## 3. Results

### 3.1. AAV Capsid Stability is pH Dependent

**Figure 1:**
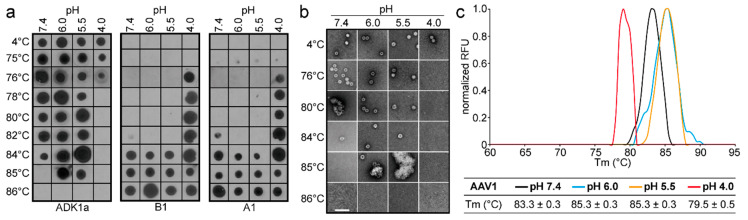
Adeno-associated virus 1 (AAV1) stability analysis. (**a**) Dot immunoblots of AAV1-green fluorescent protein (GFP) vectors at different pHs representative of physiological (7.4), early endosomal (6.0), late endosomal (5.5), and lysosomal (4.0) conditions. The virus was heat-shocked at the temperature indicated and blotted for intact capsids (ADK1a), denatured capsids (B1), and the accessibility of VP1u (A1). (**b**) Negative-stain electron microscopy (EM) of AAV1-GFP vectors treated as in (**a**). Scale bar: 100 nm. (**c**) T_m_ of AAV1-VLPs determined by DSF at the pH conditions used in (**a**) and (**b**). A thermal profile for each pH condition is shown as normalized relative fluorescence units (RFUs) versus temperature (in °C). All experiments were performed in triplicate.

Monitoring the stability of the AAV capsid in response to acidity changes was aimed at understanding how the low pH within endosomes and lysosomes prime the AAV capsid for VP1u externalization and genome uncoating. The pH-dependent thermostability of AAV1-GFP vectors assessed by native dot immunoblots showed that the capsids remained intact for up to 84 °C at pH 7.4. Negative-stain EM (EM) images confirmed this observation with intact capsids seen at 84 °C but not at 85 °C (Figure 1a,b). However, the observation of B1 and A1 signals at 84 °C suggests that some of the capsids were either already partially denatured at this temperature, consistent with the fewer intact capsids observed by EM at 84 °C compared to lower temperatures, or capsids are sufficiently “opened up” to expose the N- and C-terminus. As the pH decreased to 6.0 and 5.5, the stability of the capsids increased, with intact capsids detected at 85 °C (Figure 1a,b). At pH 4.0, the AAV1 capsids were destabilized, reducing the temperature at which the ADK1a signal was detected to 76 °C (Figure 1a). However, no capsids were observed by EM at 76 °C (although virus aggregates were present at 75 °C; data not shown), suggesting that the virus is no longer intact at this temperature (Figure 1b). The T_m_ determined for AAV1 at the different pHs by DSF (Figure 1c) largely confirmed the data from the native dot immunoblot and EM data (Figure 1a,b) and indicated that the capsids should be intact up to 83.3, 85.3, 85.3, and 79.5 °C at pH 7.4, 6.0, 5.5, and 4.0, respectively (Figure 1). A minor discrepancy was observed at pH 4.0, with the T_m_~3–4 °C higher by DSF compared to the native dot immunoblot and EM data. However, the overall trend in stability in response to pH was the same, with the highest stability occurring at pH 5.5 and the lowest at pH 4.0. In addition, the dot immunoblots showed no A1 or B1 signals under the acidic conditions at 4 or 75 °C; only heat resulted in detection at temperatures close to the T_m_ under these conditions (Figure 1a).

**Figure 2:**
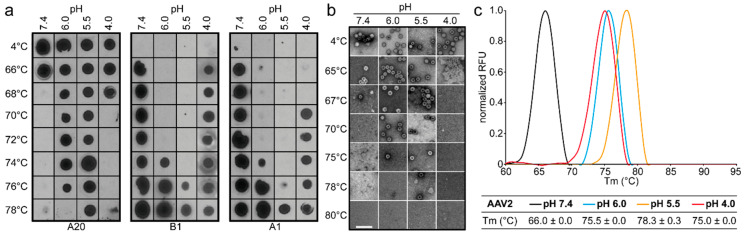
AAV2 stability analysis. (**a**) Dot immunoblots of AAV2-GFP vectors at different pHs representative of physiological (7.4), early endosomal (6.0), late endosomal (5.5), and lysosomal (4.0) conditions. The virus was heat-shocked at the temperature indicated and blotted for intact capsids (A20), denatured capsids (B1), and the accessibility of VP1u (A1). (**b**) Negative-stain EM of AAV2-VLPs treated as in (**a**). Scale bar: 100 nm. (**c**) T_m_ of AAV2-VLPs determined by DSF at the pH conditions used in (**a**) and (**b**). A thermal profile for each pH condition is shown as normalized relative fluorescence units (RFUs) versus temperature (in °C). All experiments were performed in triplicate.

The AAV2 pH-dependent thermostability profile displayed a similar trend to the AAV1 data, except that the native dot immunoblot and DSF results showed higher stability at pH 4.0 than 7.4, an observation not corroborated by the negative stain examination (Figure 2). At pH 7.4, dot immunoblots, EMs, and DSF data for AAV2 showed that the capsids were intact to 66–67 °C (Figure 2). The stability increased as the pH decreased to 6.0 and 5.5. The capsids remained intact to 75–76 °C at pH 6.0 and ~78 °C at pH 5.5, an increase of >10 °C compared to pH 7.4. Similar to AAV1, the stability of these capsids decreased at pH 4.0 relative to pH 6 and 5.5. However, surprisingly, no capsids were observed by EM at 67 °C in pH 4.0 (Figure 2b), a temperature at which both the dot immunoblot and DSF (profile peak = 50% of capsids denatured) data indicated that there should still be intact capsids (Figure 2a,c). Similar to AAV1, there was a lack of A1 and B1 detection under acidic conditions without heat (Figure 2a).

**Figure 3:**
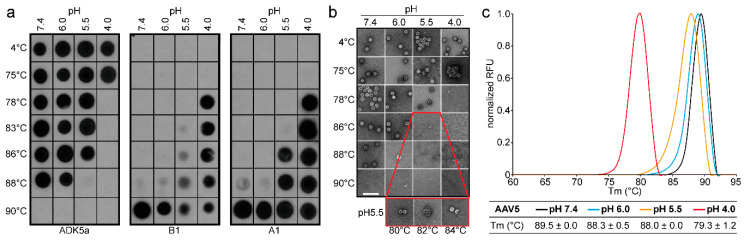
AAV5 stability analysis. (**a**) Dot immunoblots of AAV5-GFP vectors at different pHs representative of physiological (7.4), early endosomal (6.0), late endosomal (5.5), and lysosomal (4.0) conditions. The virus was heat-shocked at the temperature indicated and blotted for intact capsids (ADK5a), denatured capsids (B1), and the accessibility of VP1u (A1). (**b**) Negative-stain EM of AAV5-GFP vectors treated as in (**a**). Scale bar: 100 nm. (**c**) T_m_ of AAV5-VLPs determined by DSF at the pH conditions used in (**a**) and (**b**). A thermal profile for each pH condition is shown as normalized relative fluorescence units (RFUs) versus temperature (in °C). All experiments were performed in triplicate.

The AAV5 capsids displayed the highest T_m_ of the AAV serotypes assayed and a trend which was different to AAV1 and AAV2 (and AAV8 below). At pH 7.4, intact capsids were detected by dot immunoblot and observed by EM at 88 °C but not by 90 °C (Figure 3a,b). These results agree with the DSF data which showed that AAV5 had a T_m_ of ~89.5 °C (Figure 3c). All three methods were in agreement that the capsids maintained this extreme thermal stability at pH 6.0 (Figure 3). At pH5.5, the T_m_ of AAV5 capsids is slightly reduced to ~86–88 °C, as indicated by native dot immunoblots and DSF (Figure 3a,c). However, capsids were not detected at ~84 °C by EM, and broken capsids were clearly visible at this temperature (Figure 3b). This phenotype is in contrast to AAV1 and AAV2, as well as AAV8 (see below), with capsids being more stable at pH 5.5 than at pH 7.4. By pH 4.0, the capsids destabilized further and remained intact only up to ~76–79 °C (Figure 3). The stability of this serotype warranted further investigation and the stability under extremely acidic conditions was assayed. Significantly, negative-stain EM at pH 2.6 showed intact capsids and DSF suggested stability up to 67 °C (data not shown). Thus, the AAV5 capsids displayed a different trend in stability in response to the low pH compared to the other serotypes. The three independent methods used validated this observation. No A1 and B1 signals were detected at 4 °C without prior heating (Figure 3a). Both signals were always detected together.

**Figure 4:**
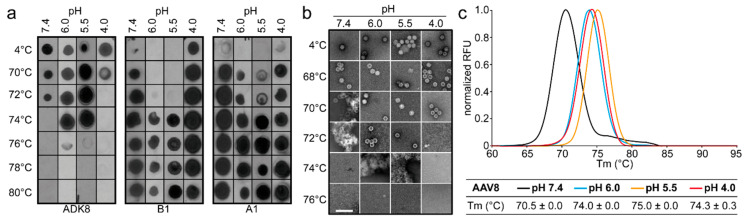
AAV8 stability analysis. (**a**) Dot immunoblots of AAV8-GFP vectors at different pHs representative of physiological (7.4), early endosomal (6.0), late endosomal (5.5), and lysosomal (4.0) conditions. The virus was heat-shocked at the temperature indicated and blotted for intact capsids (ADK8), denatured capsids (B1), and the accessibility of VP1u (A1). (**b**) Negative-stain EM of AAV8-GFP vectors treated as in (**a**). Scale bar: 100 nm. (**c**) T_m_ of AAV8 VLPs determined by DSF at the pH conditions used in (**a**) and (**b**). A thermal profile for each pH condition is shown as normalized relative fluorescence units (RFUs) versus temperature (in °C). All experiments were performed in triplicate.

At pH 7.4, the observed thermostability for AAV8 using dot immunoblots, EMs, and DSF indicated that the AAV8 capsids were intact to 70.5–72 °C (Figure 4). The capsids were stable to 74–75 °C at pH 6.0 and 5.5 (Figure 4). Dot immunoblot and EM studies at pH 4.0 showed that the capsids were intact to ~70 °C (Figure 4a,b); however, DSF indicated stability to 74.3 °C (Figure 4c). This discrepancy of a higher T_m_ at pH4.0, determined by DSF, is also seen for AAV1 and AAV2 (Figure 1 and Figure 2). Unlike AAV1, AAV2, and AAV5, the B1 signal was detected at 4 °C at pH 4.0, suggesting that there are either partially broken capsids in the sample or that rearrangements occurring at a low pH [6] expose this epitope (Figure 4a). However, the A1 epitope was detected at lower temperatures at pH 6.0 and 5.5, suggesting that the VP1u was surface-exposed before the capsids denatured, unlike the observation for the other serotypes.

### 3.2. AAVs Modify Liposomes When Heated to Externalize VP1u and at pH 5.5

**Figure 5:**
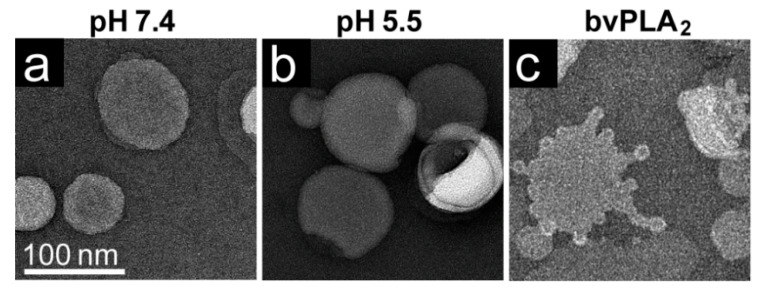
POPC liposome remodeling in the presence of PLA_2_. Liposomes (**a**) at pH 7.4, (**b**) pH 5.5, and (**c**) in presence of bee venom PLA_2_ (bvPLA_2_) at pH 7.4. Bleb formation of POPC liposomes was observed in (**c**). All experiments were performed in triplicate.

The effect of the AAV viral PLA_2_ enzymatic activity on POPC liposomes was visualized by negative-stain EM. This phospholipid was selected for this study because it accounts for >50% of all the lipids found in eukaryotic membranes: ~40% in plasma membranes, and ~50% in the late endosome and Golgi [32]. Liposomes prepared at pH 7.4 and 5.5 were round and smooth without any surface modification (Figure 5a,b). No remodeling was observed at a low pH, showing that acidic buffer alone does not induce bleb formation (Figure 5b). As a positive control, to observe the effect of a secretory PLA_2_ on POPC, bee venom PLA_2_ incubated with the liposomes showed positive curvature remodeling, as indicated by blebs extending from the liposome surface (Figure 5c).

**Figure 6:**
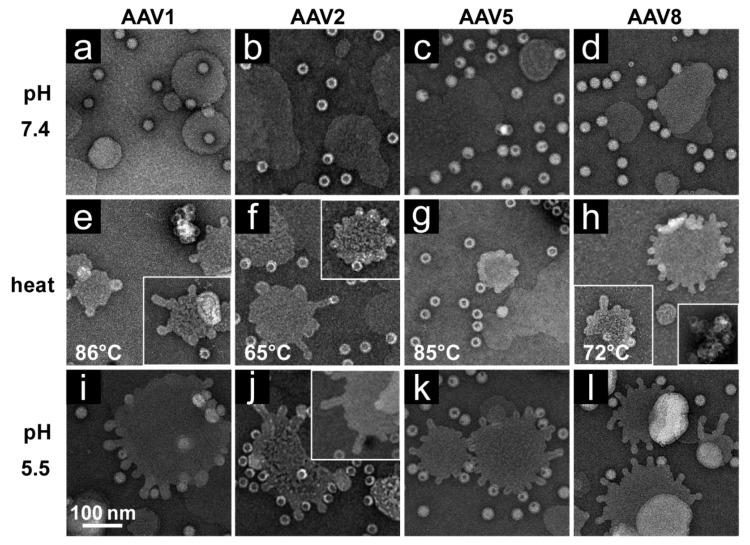
AAV–liposome interactions. POPC liposomes in presence of (**a**) rAAV1-GFP, (**b**) AAV2-VLPs, (**c**) rAAV5-GFP, and (**d**) rAAV8-GFP at pH 7.4. POPC liposomes in presence of (**e**) rAAV1-GFP, (**f**) AAV2-VLPs, (**g**) rAAV5-GFP, and (**h**) rAAV8-GFP that were heat-shocked prior to addition to liposomes. POPC liposomes in presence of (**i**) rAAV1-GFP, (**j**) AAV2-VLPs, (**k**) rAAV5-GFP, and (**l**) rAAV8-GFP that were incubated at pH 5.5 prior to addition to liposomes. Bleb formation of POPC liposomes was observed in (**e**–**l**). All experiments were performed in triplicate.

POPC liposomes incubated with untreated rAAV1-GFP, rAAV2-VLP, rAAV5-GFP, and rAAV8-GFP samples at pH 7.4 remained smooth-edged and showed no sign of remodeling (Figure 6a–d). This observation was anticipated, as the PLA_2_ domain is not expected to be externalized under neutral conditions. Using heat as a surrogate to externalize the VP1u, the serotypes were heat-treated (at temperatures observed to externalize VP1u, while some capsids remained intact (Figure 1a, Figure 2a, Figure 3a and Figure 4a) then cooled to RT prior to incubation with liposomes. Under these conditions, POPC liposomes showed successful modification by AAV1, AAV2, and AAV8 capsids (Figure 6e,f,h). Heat-treated AAV5 capsids only induced mild ruffling of the liposome membrane (Figure 6g) consistent with the reduced incorporation of VP1 into rAAV5 produced using the baculovirus/*Sf*9 system compared to rAAV5 produced in HEK293 cells [26,28]. These results confirm that the PLA_2_ within the VP1u is externalized and able to act on the liposome membranes. When the viruses were treated at pH 5.5 (no heat) and then incubated with liposomes, the four serotypes induced robust liposome modification (Figure 6i–l). This observation was unexpected because the A1 signal, which has been used previously to signify VP1u externalization [5,16,33], was not detected at pH 5.5 alone without heat for any of the serotypes in the stability tests using dot-blots (Figure 1, Figure 2, Figure 3 and Figure 4).

**Figure 7:**
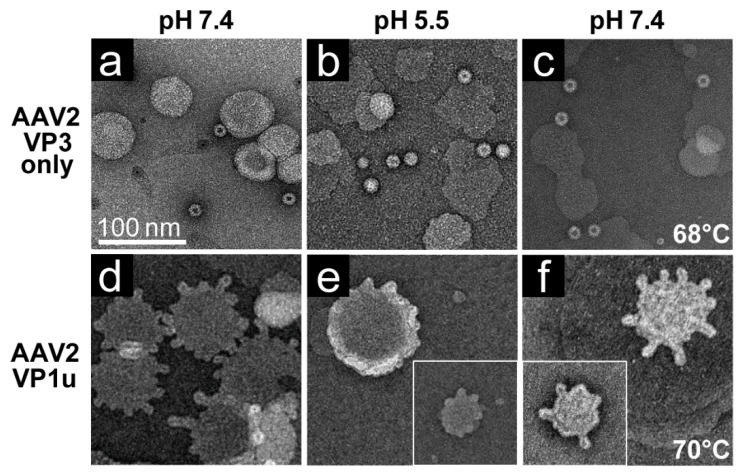
POPC liposome modification in the presence of AAV. Liposomes in presence of (**a**) AAV2 VP3 only VLPs at pH 7.4, (**b**) at pH 5.5, and (**c**) in presence of AAV2 VP3 only VLPs that were heat-shocked prior to addition to liposomes. POPC liposomes in presence of the AAV2 VP1u polypeptide at (**d**) pH 7.4, (**e**) at pH 5.5, and (**f**) after heat-shock to 70 °C prior to addition to liposomes. Bleb formation of POPC liposomes was observed in (**d**–**f**). All experiments were performed in triplicate.

To verify that the VP1u region was responsible for the observed liposome modification at pH 5.5 and after heat-treatment, AAV2 capsids comprised of only VP3 were tested for activity against liposomes. No modification was observed under neutral conditions, acidic pH conditions, or after heat-treatment (Figure 7a–c). In contrast, purified AAV2 VP1u polyprotein incubated with POPC liposomes under neutral conditions, at pH 5.5, or after heat-treatment at 70 °C, resulted in liposome modification under all three conditions (Figure 7d–f). However, as previously reported, the effect was reduced at pH 5.5 compared to pH 7.4. These findings show that the VP1u is the region of the capsids involved in inducing the lipid remodeling observed, and not the VP3 common region. The AAV2 VP1u data also show that the PLA_2_ remains active after heat-treatment and validates the previous use of heat as a surrogate for externalizing the VP1u from capsids for PLA_2_ assays.

### 3.3. AAVs Show a Temperature and pH Dependence of Transduction

**Figure 8:**
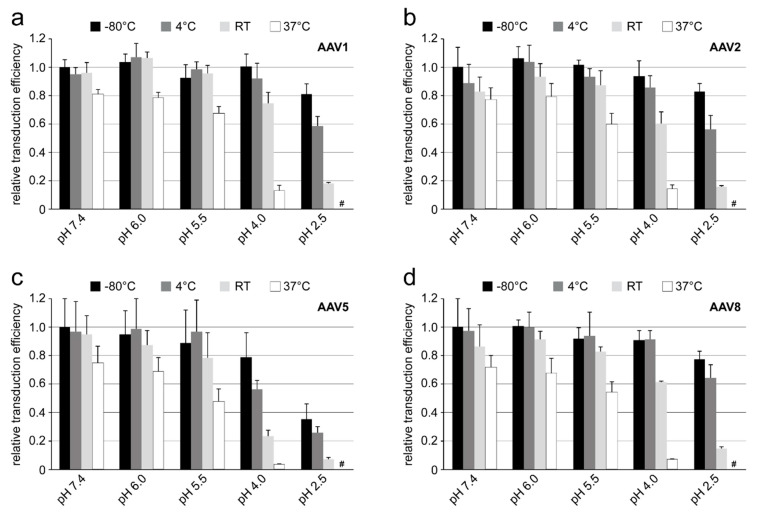
Effect of pH and temperature on AAV transduction. Transduction efficiency of (**a**) rAAV1, (**b**) rAAV2, (**c**) rAAV5, and (**d**) rAAV8 (all packaging the luciferase gene) in HEK293 cells infected with virus incubated for 24 h in citrate-phosphate buffer at the indicated pH and storage temperature. The transduction efficiency for each AAV serotype is shown relative to virus stored at pH 7.4 and −80 °C. The experiments were performed in triplicate and are displayed as mean+SD (n = 3). #: luciferase read-out similar to uninfected control cells (data not shown) which indicates no transduction.

Cellular transduction by rAAV-Luciferase vectors following a 24 h pre-incubation at different pHs (7.4, 6.0, 5.5, 4.0, and 2.5) and temperatures (−80 °C, 4 °C, RT, and 37 °C) showed a similar trend in transduction efficiency for all AAV serotypes (Figure 8). The highest transduction level was observed at pH 7.4 and 6.0 for the samples stored at −80 and 4 °C. Incubation of the vectors at 37 °C negatively impacted transduction efficiency at a neutral pH by 20 to 30%. A higher temperature combined with low pH storage conditions such as pH 4.0 reduced transduction efficiency by ~80 to 90%. Compared to rAAV1, rAAV2, and rAAV8 (Figure 8a–c), rAAV5 was the most susceptible to low pH storage conditions (Figure 8c). Transduction efficiency decreased by ~80% when the vectors were exposed to pH 4.0 at RT for 24 h. AAV1, AAV2, and AAV8 vectors maintain most of their infectivity (reduction in transduction efficiency of less than 40%) at the same conditions. At pH 2.5, used to recapitulate extracellular intestinal conditions, all the viruses lost transduction efficiency by at least 20% when stored at −80 °C (Figure 8). Higher storage temperatures led to further loss of transduction efficiency, with no detectable transduction for samples pre-incubated at 37 °C for 24 h, regardless of AAV used.

## 4. Discussion

**Figure 9:**
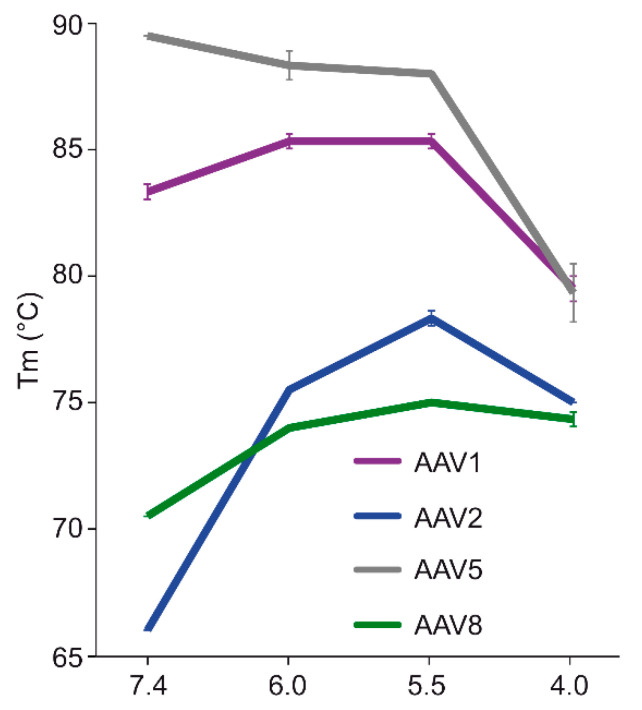
Melting temperature of AAV capsids under different pH conditions. Plot of the T_m_ for AAV1, AAV2, AAV5, and AAV8 at physiologic (7.4), early endosome (6.0), late endosome (5.), and lysosome (4.0) conditions. The experiments were performed in triplicate and are displayed as mean+SD (n = 3).

The comparative analysis of AAV1, AAV2, AAV5, and AAV8 confirmed differences in their thermostability, with AAV5 being 20 °C more stable than AAV2 (Figure 9), as previously reported [34,35]. Despite the large range of T_m_ values for the four serotypes at physiological pH, with the exception of AAV5, acidification reminiscent of the early and late endosomes increased stability, while lysosomal pH conditions decreased the stability below that at pH 7.4 for AAV1 (Figure 9). For AAV2 and AAV8, stability was lowest at pH 7.4. The commonality in stability trend as pH drops suggests that the dynamics associated with trafficking through the endosome is the same for AAV1, AAV2, and AAV8, and suggests a conserved mechanism for VP1u externalization and/or readiness for uncoating. AAV5 is the only serotype analyzed to date which showed a continuous decrease in stability with acidification (Figure 9). This observation was recapitulated with differential scanning calorimetry (DSC, data not shown). AAV5 is also the most thermally stable serotype [35] tested to date. AAV5 has been shown to utilize multiple cellular entry pathways. Thus, its enhanced stability and difference in pH stability profile may be important for trafficking through an alternative pathway. Changing pH alters the protonation state of amino acids, which could alter the interactions between residues or induce conformational changes. Such changes could confer altered stability. For AAV8, structures determined under endo/lysosomal conditions provided information on side-chain conformational changes within and between monomers associated with altered protonation states, including histidines [6]. These changes in interacting residues could influence thermostability.

Each of the four AAV serotypes analyzed exhibited a transduction efficiency that followed the same trend as their pH-dependent stability when stored at −80 and 4 °C (Figure 1, Figure 2, Figure 3 and Figure 4 and Figure 8). Infectivity was maintained at pHs 7.4, 6.0, and 5.5, but was significantly reduced at pH 4.0 for all the serotypes, the conditions at which AAV1 and AAV5 were the least stable and the stability was dropping for AAV2 and AAV8 compared to pH 5.5 (Figure 9). The most significant drops in infectivity were observed at pH 4.0 and 2.5, and 37 °C. This reflects the conditions that the AAVs encounter after oral administration. However, although successful gene expression in mice has been reported for AAV vectors after oral administration [36,37], the transduction results here highlight the necessity to minimize the exposure time to low-pH and high-temperature conditions to preserve long-term AAV infectivity. This includes during different AAV purification techniques, e.g., nanobody affinity chromatography [38], iodixanol gradient centrifugation [27], or low pH flocculation [39], utilizing acidic reagents that come into contact with the virus.

Similar comparative stability and infectivity studies have been performed for other parvoviruses, e.g., MVM, human parvovirus B19, and AAV9. MVM was found to be stable to ~75 °C at pH 7.5 [40]. The MVM capsid stability is not reduced at pH 4.0 (unpublished data, not shown) and the capsids do not show a reduction in infectivity at this pH, in contrast to what has been observed here or reported for the other AAVs [41]. MVM capsids were found to destabilize in response to pH 2.7, although these capsids still displayed high thermal stability, with a T_m_ of ~60 °C [40]. Infectivity was not tested at this pH. B19, which shows relatively low stability compared to the other parvoviruses, with a T_m_ of ~60 °C at pH 7.5 [42], exhibits reduced stability as well as infectivity at pH 4.0 [41]. For AAV9, exposure of capsids to acidic pH for a short period of time (2 h) at RT did not reduce its infectivity. However, after 24 h, a 10-fold reduction in infectivity was observed for capsids incubated at pHs between 5–6 compared to pH 7.5 [39]. Incubation at pHs below 5 did not reduce infectivity for AAV9. Interestingly, AAV9 was also more stable at pHs 5 and 6, as observed here for AAV1, AAV2, and AAV8. Thus, the parvoviruses exhibit different thermostabilities and likely do not have a generalized correlation of capsid stability due to their mechanism of infectivity.

The AAV VP1u (aa 1 to 137, AAV2 numbering), containing the PLA_2_ motif (75-HDXXY-79; AAV2 numbering), is essential for host infection. One possible explanation for why liposomes incubated with AAV at pH 5.5 showed blebbing indicative of PLA_2_ action (Figure 6) while the A1 epitope (123-KRVLEPLGL-131) was not detected under the same condition by dot immunoblot (Figure 1, Figure 2, Figure 3 and Figure 4), is that the A1 epitope is not surface-exposed when the PLA_2_ motif is. It is feasible that the N-terminally located PLA_2_ motif is able to externalize from capsids under low pH conditions but the C-terminally located A1 epitope within VP1u is not. This would allow for liposome modification while preventing detection of the VP1u by dot-blot. Another possible explanation is that capsid contact with lipids is required for VP1u externalization at a low pH in the absence of heat as a surrogate. The data presented here (Figure 5, Figure 6 and Figure 7) provide the first evidence that lipids may be required for externalization of the VP1u domain at low pH. No modification was observed with the VP3-only capsids, while lipid remodeling was seen with the purified VP1u-only polypeptide (Figure 7). These observations confirm that residues within the VP1u region are responsible for the observed blebbing and not residues within the common VP1/2 or VP3 regions.

Structural re-arrangement of the VP1u region has been proposed for AAVs, with unfolding at low pH (5.5 and 4.0), followed by refolding if conditions are returned to a physiological pH, based on circular dichroism (CD) analysis [43]. The blebbing at pH 5.5 for the four serotypes tested, is consistent with a functional PLA_2_, suggesting that the VP1u has re-folded to a functional state in the presence of liposomes after externalization from the interior of the capsid. Liposome modification following heat shock at temperatures where the A1 epitope is detected for each AAV serotype at physiological pH is consistent with VP1u externalization or broken capsids. The lack of liposome modification by the AAVs under physiological pH (Figure 6) without heating is consistent with the VP1u being located inside capsids [43]. The ability of the AAV2 VP1u polypeptide alone to modify liposomes following heating validates the use of heat as a surrogate for monitoring VP1u externalization and PLA_2_ function. Significantly, the functional pH for PLA_2_ activity of the AAV capsids and the AAV2 VP1u alone were 5.5 and 7.5, respectively. The AAV2 VP1u-alone activity is consistent with previous reports for expressed protein alone [12]. However, the low-pH-induced PLA_2_ function in capsids is more consistent with its role in endo/lysosomal escape. Interestingly, canine parvovirus (CPV), an autonomous member of the *Parvoviridae*, belonging to the *Protoparvovirus* genus, has been reported to undergo a structural change at low pH in response to lipids [44]. Furthermore, CPV has been shown to disrupt cholesterol-lipid membranes under acidic pH 5.5 conditions but not at neutral pH [45]. It was also reported that CPV capsids without the VP1u domain were unable to perform this modification, as observed for the VP3-only AAV2 capsids. These similarities in phenotype show the conservation of parvovirus functions, including rearrangements to externalize VP1u during trafficking en route to the nucleus for genome replication.

Interestingly, liposome blebbing was observed without direct membrane-associated viral capsids (Figure 6). This raises the possibility that after the VP1u has been externalized from capsids, some are cleaved to interact with the bilayer to perform the membrane modification. The AAVs have also been reported to contain internal and external protease functions [3,46]. The VP1u region was shown to exhibit proteolytic activity, capable of digesting disordered proteins [46]. In addition, protease sites have been identified in the VP1u, and several of these sites occur between the PLA_2_ domain and the A1-binding epitope [34]. Thus, it is possible that the VP1u is cleaved from some capsids, at an amino acid position between the PLA_2_ motif and A1 epitope, during endosomal trafficking, to act on membranes. This would be consistent with our inability to detect the A1 epitope in liposome-treated capsids, even at pH 5.5 (data not shown). This hypothesis partially contrasts previous reports stating that, for MVM, VP1u remains tethered to the capsid during trafficking [47]. Thus, this cleavage possibility requires further study.

The three techniques utilized to probe the stability of the AAV serotypes agree on the stability profile for each serotype, with only small differences in the observed T_m_. However, the reason why AAVs are more stable at pH 6.0 and 5.5 is unknown. Small-angle neutron scattering (SANS) analysis of rAAV8 capsids at pH 5.5 shows that the packaged DNA compresses by ~3 Å (Venkatakrishnan, McKenna, and Agbandje-McKenna, unpublished data). This compression results in a separation of genome and capsids, which could play a role in enabling genome release or externalization of the VP1u. Structural studies of AAV8 at pHs 7.4, 6.0, 5.5, and 4.0 showed pH-dependent VP conformational rearrangements and reversible loss of DNA density as pH drops [6]. This reversibility indicates that the DNA density was either compressed or became disordered due to lack of VP interactions at low pH and left a gap between the capsid interior and packaged genome. Furthermore, in DNA-filled AAV capsids, a space exists under the icosahedral 5-fold axes [48]. Evidence suggests that the viral genome is ejected from intact parvovirus capsids in a 3′-5′ direction via the 5-fold channel in parvovirus MVM and the AAVs [49,50]. However, unlike the PLA_2_ domain, release of the packaged genome does not occur during endosomal trafficking. In fact, acidic pH has been shown to suppress genome release for AAV2, and suggests that the required structural changes for genome release and VP1u externalization are distinct events [49]. This suggests that the alterations to capsid stability for AAV8 and genome rearrangement seen in response to acidic pH [6] are likely not related to preparation of genome release but rather VP1u externalization. As previously stated, CD analysis of AAV1 led to the prediction that VP1u loses its α–helical secondary structure under acidic conditions and is unfolded by pH 5.5 [43]. This action was reversible with the VP1u refolding upon return of the assay study condition to pH 7.5. The space under the 5-fold axis may be required for this unfolding process for threading of the VP1u through the channel to interact and modify liposomes at a low pH. Thus, future structural studies of heated capsids and additional structures of capsids exposed to a low pH after liposome exposure will be informative.

## 5. Conclusions

While AAVs have been reported in lysosomes, they typically escape from either the early or late endosome [16,17,18]. As such, assays at pH 6.0 and 5.5 are more biologically relevant for determining structural and stability changes associated with endosomal pH transitions. Our stability and liposome assays demonstrate that AAV capsids are dynamic, and trafficking through endo-/lysosomal conditions alters their physical properties. These studies also suggest a role for storage pH and temperatures in affecting the externalization of VP1u and capsid disassembly. Significantly, this study shows that pH 5.5 is able to release/externalize the VP1u for its PLA_2_ function on lipid membranes. The data provide information that will inform future studies, including 3D structure determination, aimed at further understanding of AAV trafficking determinants.
